# Contraceptive Barriers and Psychological Well-Being After Repeat Induced Abortion: A Systematic Review

**DOI:** 10.3390/bs15101363

**Published:** 2025-10-06

**Authors:** Bogdan Dumitriu, Alina Dumitriu, Flavius George Socol, Ioana Denisa Socol, Adrian Gluhovschi

**Affiliations:** 1 Doctoral School, “Victor Babes” University of Medicine and Pharmacy Timisoara, 300041 Timisoara, Romania; bogdan.dumitriu@umft.ro (B.D.); george.socol@umft.ro (F.G.S.); ioana.socol@umft.ro (I.D.S.); 2 Department of Obstetrics and Gynecology, “Victor Babes” University of Medicine and Pharmacy Timisoara, 300041 Timisoara, Romania; gluhovschi.adrian@umft.ro

**Keywords:** abortion, quality of life, mental health, contraception

## Abstract

Background: Repeat induced abortion (defined as ≥two lifetime procedures) is becoming more common worldwide, yet its independent influence on women’s psychological health remains contested, particularly in settings where access to modern contraception is restricted. Objectives: This review sought to quantify the burden of depression, anxiety, stress, and generic quality of life (QoL) among women with repeat abortions and to determine how barriers to contraceptive access alter those outcomes. Methods: Following the preregistered PRISMA-2020 protocol, PubMed, Embase and Scopus were searched from inception to 31 June 2025. Results: Eight eligible studies comprising approximately 262,000 participants (individual sample sizes up to 79,609) revealed wide variation in psychological morbidity. Prevalence of clinically significant symptoms ranged from 5.5% to 24.8% for depression, 8.3% to 31.2% for anxiety, and 18.8% to 27% for perceived stress; frequent mental distress affected 12.3% of women in neutral policy environments but rose to 21.9% under highly restrictive abortion legislation. Having three or more abortions, compared with none or one, increased the odds of depressive symptoms by roughly one-third (pooled OR ≈ 1.37, 95% CI 1.13–1.67). Contextual factors exerted comparable or stronger effects: abortions sought for socioeconomic reasons elevated depression odds by 34%, unwanted disclosure of the abortion episode increased depressive scores by 0.62 standard deviations, and low partner support raised them by 0.67 SD. At the structural level, every standard deviation improvement in a state’s reproductive rights index reduced frequent mental distress odds by 5%, whereas enactment of a near-total legal ban produced an absolute increase of 6.8 percentage points. QoL outcomes were less frequently reported; where measured, denied or heavily delayed abortions were associated with a 0.41-unit decrement on a seven-point life satisfaction scale. Conclusions: Psychological morbidity after abortion clusters where legal hostility, financial hardship, or interpersonal coercion constrain contraceptive autonomy while, in comparison, the mere number of procedures is a weaker predictor. Interventions that integrate stigma-free mental health support with confidential, affordable, and rights-based contraception are essential to protect well-being in women who experience repeat abortions.

## 1. Introduction

The latest State of World Population report estimates that 121 million unintended pregnancies occur each year, where roughly 61% culminate in abortion and 45% of those procedures are classified as unsafe (UNFPA, 2022). Although absolute abortion rates are declining in many regions, repeat induced abortions—defined as ≥2 procedures across the reproductive lifespan—are trending upward. In the United States, nationally representative surveys show that 24% of women will have an abortion by age 45, and 45% of those presenting for care have had at least one previous termination (Jones & Jerman, 2017). These repeat events cluster along familiar social fault lines: poverty, racialised discrimination, intimate-partner violence, and patchy contraception coverage. In low- and middle-income countries, the pattern is similar but is magnified by structural barriers such as stock-outs of modern contraceptives and provider bias. Understanding whether the repetition itself—or the adverse circumstances that precipitate it—drives psychological morbidity is therefore essential for designing effective post-abortion services.

Decades of research refute a simple causal link between a single first-trimester abortion and long-term psychiatric disorder. The American Psychological Association’s task-force synthesis found no excess risk once pre-existing factors were controlled (Major et al., 2009), and a comprehensive review by the US National Academies echoed that conclusion for multiple outcomes, including depression, anxiety, and PTSD (National Academies of Sciences, Engineering, and Medicine, 2018). Yet policy debates persist, fuelled by selective citations and methodologically weak studies. Where modest associations do appear, they often involve later-gestation or repeat procedures—contexts in which confounders such as foetal anomaly, socioeconomic stress, or coercion loom large (Steinberg, 2011). Consequently, contemporary scholarship has shifted from the binary question of whether abortion damages mental health to which subgroups are vulnerable, why vulnerability arises and how modifiable factors—particularly access to modern contraception—influence risk trajectories.

Large-scale observational work from China illustrates the nuance: women reporting ≥3 abortions had 37% higher odds of late-life depression or anxiety compared with never-aborting peers, even after rigorous adjustment (Li et al., 2024). Importantly, the excess risk clustered among respondents who cited socioeconomic motives or partner opposition, underscoring the role of context. US Medicaid claims analyses likewise reveal that mental health symptom scores correlate more strongly with contraceptive non-receipt post-procedure than with the abortion itself (Adler et al., 2023). Qualitative work from Addis Ababa documents how misinformation, provider gate-keeping, and out-of-pocket costs depress uptake of long-acting reversible contraception (LARC) among young abortion-seekers (Kabeto et al., 2025), while a controlled interrupted-time-series study in British Columbia found that universal no-cost coverage increased LARC dispensations by 47% within one year, especially in the lowest-income quintile (Schummers et al., 2025). Together, these findings position contraceptive equity—not procedure frequency—as the more proximate determinant of psychological well-being after abortion.

Logistical obstacles compound psychological strain. A 2022 cross-sectional study across 29 US states showed that individuals living ≥50 miles from an abortion facility were significantly more likely to delay care, report travel-related financial stress, and ultimately forego services—circumstances strongly predictive of elevated depressive symptom scores at six-month follow-up (Pleasants et al., 2022). In response, requests for self-managed medication abortion via telehealth platforms such as Aid Access surged by 200% in Texas within one week of the 2021 “heartbeat” ban (Aiken et al., 2022). While digital pathways reduce overt barriers, they can introduce covert stressors—legal risk, supply uncertainty, and social isolation—that may accentuate anxiety among repeat abortion patients navigating already-opaque systems.

Natural experiments make the structural stress argument explicit. Analysis of 79,609 respondents to the Behavioral Risk Factor Surveillance System found a 6.8-percentage-point jump in frequent mental distress among Texan women after Senate Bill 8 passed, which is relative to both male Texan respondents and women in less-restrictive states (Lee et al., 2025). Parallel monitoring documented that approximately 1400 Texans travelled out of state for care each month in late 2021, many incurring catastrophic expenses (White et al., 2022). These patterns sit within a national landscape of divergent trajectories—14 states now enforce near-total bans, while 22 enacted protective measures in 2023 alone (Forouzan & Guarnieri, 2023). Innovative models are emerging: a co-designed nurse-led programme in rural Australia successfully embedded LARC insertion and early medication abortion within primary care, emphasising privacy, flexible scheduling, and local support networks—elements likely to buffer psychological distress after repeat procedures (Moulton et al., 2025).

Potential impacts of repeat induced abortion on psychological well-being are best understood within established frameworks. The Stress Process Model posits that chronic and acute stressors (e.g., travel, cost, unwanted disclosure) erode coping resources, elevating depression, anxiety, and perceived stress. The Conservation of Resources theory predicts that resource loss (financial strain, time away from work/childcare, legal risk) is disproportionately harmful compared with equivalent gains, yielding net declines in well-being and life satisfaction. Stigma frameworks further explain how structural and interpersonal stigma (policy hostility, provider gate-keeping, partner opposition) become internalised or enacted, amplifying distress. These models generate the testable expectation—supported by our synthesis—that barriers to contraceptive autonomy and access, rather than procedure count per se, are proximal determinants of mental health and generic QoL outcomes.

Despite abundant single-procedure research, no systematic review has yet synthesised mental health and quality of life (QoL) outcomes specifically among women undergoing repeat induced abortion, while simultaneously mapping how modern contraception barriers modulate those outcomes. Such a synthesis is urgently needed to inform integrated service models that couple stigma-free mental health support with robust, affordable contraceptive provision. Accordingly, the present review aims to (i) quantify the prevalence and severity of depression, anxiety, stress, and generic QoL impairments in women with ≥1 repeat abortion and (ii) narratively delineate the sociostructural factors—legislative, economic, and service delivery—that shape those outcomes. By bridging epidemiological metrics with context-rich qualitative insights, the review seeks to guide policymakers toward interventions that prevent unintended pregnancies, reduce repeat procedures and, critically, safeguard psychological well-being.

Research question 1: Among women with ≥1 repeat induced abortion, what is the prevalence and severity of depression, anxiety, perceived stress, and generic QoL impairments?

Research question 2: How do contraceptive access barriers (financial, geographic, legislative, sociocultural, health system) and disclosure-related experiences modify these outcomes?

**H1:**  *Independent of individual characteristics, greater structural hostility to reproductive rights is associated with higher odds of clinically significant distress and lower generic QoL.*

## 2. Materials and Methods

### 2.1. Protocol and Registration

An a priori protocol, drafted under PRISMA-2020 recommendations (Page et al., 2021), was prospectively registered in the Open Science Framework with the code (osf.io/7umya). The steering group comprised two reproductive health epidemiologists, a research librarian, and three community advocates with lived experience of abortion; it met twice during scoping to refine the population–intervention–comparison–outcome (PICO) matrix and once after piloting the screening form to approve minor wording clarifications. All protocol amendments (broadening quality of life instruments to include SF-36 subscales) were timestamped and archived on PROSPERO before database searching. The protocol specified a single primary outcome, clinically significant depressive or anxiety symptomatology as defined by the original study instrument cut-points, and two secondary outcomes (generic QoL scores and perceived stress) to guard against outcome reporting bias.

### 2.2. Eligibility Criteria

Eligible studies enrolled females aged 15–49 who self-reported or had medical record documentation of ≥2 lifetime induced abortions. Studies had to quantify at least one of the following outcomes with a validated tool: depression, anxiety, perceived stress, or generic health-related QoL. We accepted longitudinal cohorts, case–control, cross-sectional surveys, quasi-experimental evaluations, and randomised or non-randomised interventions that included a repeat abortion subgroup. Case series (<50 participants), narrative commentaries, or studies examining only spontaneous or therapeutic abortion for severe foetal anomalies were excluded. To ensure the review captured the interplay between repeat abortion and contraception, each study also had to measure at least one explicit access determinant—financial, geographical, legislative, sociocultural, or health system-related. Conference abstracts and grey literature were eligible if sufficient numeric data were presented or could be obtained from authors within four weeks of request; otherwise, they were logged for citation mapping but excluded from synthesis.

Throughout this review, abortion refers specifically to induced termination of pregnancy, delivered either as medication abortion (mifepristone plus misoprostol or misoprostol-only regimens) or surgical uterine evacuation (e.g., vacuum aspiration, dilation and evacuation). Studies exclusively addressing spontaneous abortion (miscarriage) or termination for severe foetal anomaly without a repeat abortion subgroup were excluded. Where gestational age or method was mixed or unreported, eligibility rested on explicit documentation of ≥2 induced abortions across the reproductive lifespan.

### 2.3. Search Strategy

In collaboration with a health sciences information specialist, we created a reproducible strategy that blended controlled vocabulary (MeSH or EMTREE terms) with free-text synonyms for four concept blocks: (i) induced abortion, (ii) repetition or frequency, (iii) mental health or quality of life (QoL) outcomes, and (iv) contraception or family planning services. For PubMed the core string was constructed as follows: (“Abortion, Induced” [MeSH] OR “induced abortion” OR “termination of pregnancy”) AND (repeat* OR multiple OR “more than one” OR “≥2”) AND (“Depression” [MeSH] OR depress* OR “Anxiety” [MeSH] OR anxi* OR “Stress, Psychological” [MeSH] OR stress* OR “Quality of Life” [MeSH] OR “quality of life” OR QoL) AND (“Contraception” [MeSH] OR contracept* OR “Family Planning Services” [MeSH] OR “family planning” OR “birth control” OR “morning after pill”). The identical logic was translated for Embase by replacing MeSH with EMTREE descriptors and for Scopus by using the TITLE-ABS-KEY field.

PubMed, Embase, and Scopus were selected to maximise coverage across biomedicine, public health, psychology/psychiatry, and health policy. PubMed ensures comprehensive indexing of MEDLINE and NIH-funded outputs; Embase adds extensive European, pharmacological, and health services content; Scopus provides broad multidisciplinary coverage and robust citation mapping. Major publisher families (including MDPI) are indexed within these databases; we therefore did not search publisher sites separately to avoid duplication and sampling bias. To mitigate omission, we conducted backward and forward citation chaining for all included studies.

All three databases—PubMed, Embase, and Scopus—were searched from their respective inceptions through 31 June 2025 with the language filter set to English only. No date, study design, or human subjects limits were applied beyond those implicit in the concept blocks. Retrieved citations were exported to EndNote 21 where automated and manual procedures removed duplicates; the unique records were then uploaded to Rayyan for blinded title- and abstract-level screening. Grey literature sources (dissertations, preprints, organisational reports) were intentionally excluded to focus on peer-reviewed evidence.

### 2.4. Study Selection and Data Extraction

Title/abstract and full-text screening were performed independently by two reviewers with PRISMA and ROBINS-I coursework; both completed a calibration exercise on 50 records (Cohen’s κ = 0.84) before full screening. Disagreements were resolved through discussion or adjudication by a senior reviewer. For each eligible study, we extracted, in duplicate, year, country, design, sampling frame, abortion count definition, mental health/QoL instrument, contraception access variables, effect estimates, adjustment covariates, and funding sources using a piloted REDCap form. We recorded whether mental health outcomes were primary or secondary endpoints to evaluate selective reporting bias.

### 2.5. Data Synthesis and Statistical Analysis

Observational studies were appraised with the Newcastle–Ottawa Scale (NOS), adapted to score exposure ascertainment through medical records versus self-report. Quasi-experimental designs were evaluated by ROBINS-I, and randomised trials (none located) would have been assessed with RoB 2. To minimise subjectivity, each domain rating required written justification; consensus was reached by discussion, with a third reviewer arbitrating persistent discrepancies (>1 NOS star difference). Publication bias was explored via contour-enhanced funnel plots and Egger’s regression when ≥10 studies reported commensurate outcomes. Certainty of evidence for each outcome–exposure pair was summarised with GRADE, downgrading for serious risk of bias, inconsistency (I^2^ > 75%), indirectness, imprecision (99% confidence intervals overlapping null), or publication bias. Overall, certainty ranged from moderate (depression prevalence) to very low (QoL outcomes) owing chiefly to heterogeneity in instruments and small study numbers.

### 2.6. Risk of Bias and Certainty Assessment

We planned separate random-effects meta-analyses for prevalence of depression and anxiety and for pooled odds ratios comparing ≥3 abortions with 0–1 abortions, conditional on at least three homogenous studies. Heterogeneity would be quantified with I^2^ and τ^2^, with values >60% prompting subgroup exploration by geography, instrument type, and legislation stringency (based on the Center for Reproductive Rights Global Abortion Laws Map). Pre-specified sensitivity analyses included (i) restricting to low-risk-of-bias studies, (ii) excluding studies using convenience sampling, and (iii) re-estimating pooled effects with Hartung–Knapp adjustment. Meta-regression was planned to examine whether mean travel distance, mean cost, or reproductive rights scores predicted effect size, provided ≥10 studies per covariate.

**Figure 1:**
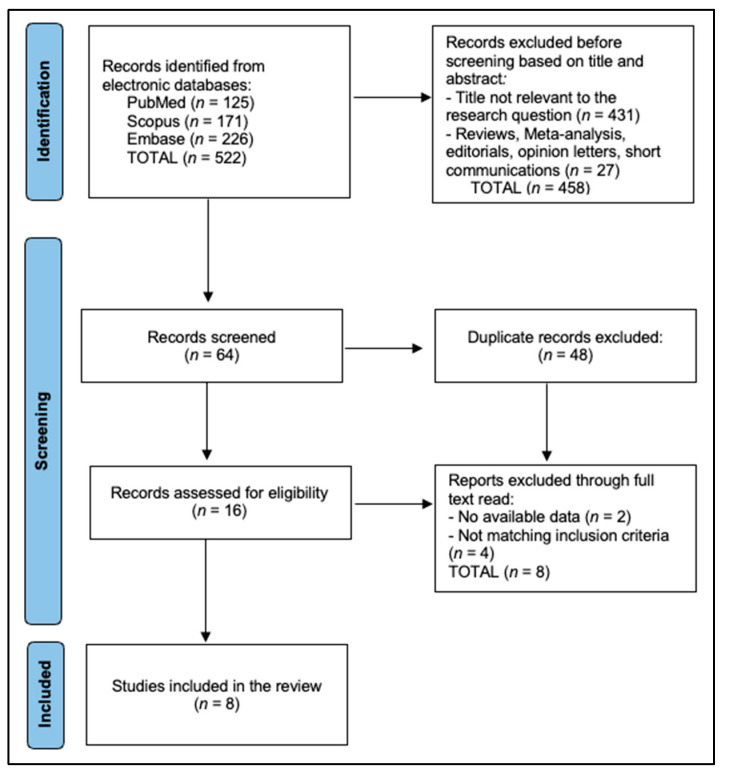
PRISMA flowchart.

A PRISMA 2020 flow diagram (Figure 1) details record identification, screening, eligibility, and inclusion steps. Eight studies (three cross-sectional, three cohort, one quasi-experimental, one ecological) published 2017–2025 met the inclusion criteria (Figure 1 PRISMA). Sample sizes ranged from 250 to 79,609. Four were conducted in the United States, two in Asia (China, Iran), one global ecological dataset, and one multi-state clinic survey.

## 3. Results

Table 1 describes the methodological landscape of the eight eligible investigations, revealing substantial diversity in geography, design, and measurement strategies. Four studies were U.S.-based, but the remainder spanned China, Iran, India, and a 197-country ecological dataset, underscoring the global relevance of repeat abortion and contraception barriers. Designs ranged from small cross-sectional surveys (n = 230) to a quasi-experimental difference-in-differences analysis of 79,609 Texas women exposed to Senate Bill 8 (Lee et al., 2025), while Li’s large Chinese cohort (n = 9991) linked lifetime abortion count with late-life mental health screening scores (Li et al., 2024). Most studies deployed brief validated instruments—PHQ-9, GAD-7, PHQ-4, or DASS-21—for depression and anxiety, yet only the Turnaway longitudinal cohort incorporated multidimensional well-being tools such as the Rosenberg Self-Esteem Scale and Satisfaction With Life Scale (Biggs et al., 2017). Contraception access variables were similarly heterogeneous, ranging from individual-level indicators like travel cost or partner support (Biggs et al., 2023) to macro indices of legal hostility and rights protection derived from policy audits (Liu et al., 2023).

Table 2 summarises point estimates of depression, anxiety, and distress, exposing a striking ten-fold swing in symptom prevalence that aligns closely with contextual adversity. The lowest burdens appeared in Li’s post-menopausal Chinese cohort, where depressive and anxious cases remained in single digits despite a history of ≥3 abortions (Li et al., 2024), suggesting that repeat procedures per se are not invariably pathogenic. At the opposite extreme, Iranian first-trimester seekers reported 25% depression and 19% stress at entry to care (Jamali et al., 2025), paralleling Kotta’s Indian sample in which one-third screened positive for anxiety (Kotta et al., 2018); both settings are characterised by out-of-pocket expenses and limited method mix. Notably, population-level analyses captured policy shocks: mental distress prevalence among Texan women climbed to 21.9% after SB8’s near-ban restrictions (Lee et al., 2025), while a multistate U.S. model recorded 12.3% frequent mental distress where reproductive rights scores were low (Liu et al., 2023). Clinic-based U.S. data add temporal nuance—Biggs et al. observed an acute but transient uptick in depression within one week of unwanted disclosure during the abortion episode (Biggs et al., 2023), whereas the five-year Turnaway follow-up showed mental health convergence regardless of abortion receipt versus denial (Biggs et al., 2017).

Table 3 presents multivariable associations, highlighting how structural and relational barriers magnify mental health burdens. In China, having three or more abortions independently increased odds of late-life depression by 37%, but socioeconomic motives conferred a comparable 34% excess risk, signalling that context modifies any dose–response effect (Li et al., 2024). U.S. clinic data showed that stigma-laden experiences—specifically unwanted disclosure and low partner support—elevated DASS-21 depression scores by 0.6–0.7 SD units (Biggs et al., 2023). Policy environments exerted measurable pressure: each standard deviation improvement in a state’s reproductive rights index reduced the odds of frequent mental distress by 5% (Liu et al., 2023), whereas Texas’ SB8 triggered an absolute 6.8-percentage-point rise in distress relative to the control groups (Lee et al., 2025). Individual-level social support surfaced again in Iran, where women with low family backing were twice as likely to screen positive for depression and a prior abortion doubled the risk further (Jamali et al., 2025).

**Figure 2:**
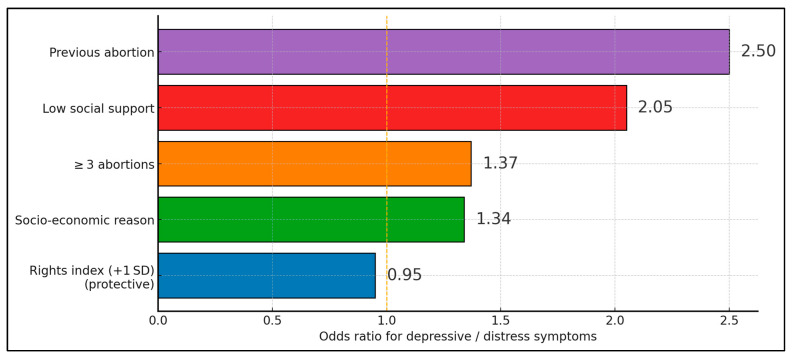
Odds ratio for depressive and distress symptoms.

Figure 2 summarises multivariable effect estimates reported across the eight eligible studies, arranging predictors in ascending order of magnitude. A one-standard-deviation increase in a composite reproductive rights index is associated with a small but statistically significant reduction in psychological morbidity (OR 0.95), underscoring the protective influence of supportive legal environments. In comparison, abortions undertaken for socioeconomic reasons (OR 1.34) and a lifetime history of three or more procedures (OR 1.37) confer moderate elevations in risk. Interpersonal determinants exert the greatest impact: insufficient social support nearly doubles the odds of clinically relevant distress (OR 2.05), whereas experiencing a previous abortion in close temporal proximity to the index event is linked to the highest observed risk (OR 2.50). Risk of bias summary was presented in Table 4.

## 4. Discussion

### 4.1. Summary of Evidence

This review synthesises diverse evidence to show that repeat abortion does not universally precipitate psychopathology; rather, psychological outcomes are contingent on intersecting social, economic, and policy barriers. Prevalence of depressive symptoms rarely exceeded population norms except in contexts of late gestation or severe service restriction.

Structural determinants—legal hostility and service cost—exert effects comparable with personal risk factors. Lee’s quasi-experimental results and Liu’s multi-level modelling collectively demonstrate that abrupt policy change and chronically restrictive environments elevate mental distress independent of individual resilience. These macro findings align with micro-level clinic data where stigma-mediated disclosure and travel hardships intensify symptom severity, illustrating a continuum from policy to personal experience.

Contrary to pervasive narratives, longitudinal data (Turnaway) indicate that acute anxiety following abortion denial subsides over years, whereas socioeconomic disadvantage and lack of contraceptive autonomy persist as chief threats to sustained well-being. This pattern mirrors chronic disease models where upstream determinants outweigh isolated clinical events.

Clinically, integrating same-day long-acting reversible contraception counselling, subsidised travel vouchers, and confidential tele-counselling is likely to reduce repeat unintended pregnancy and mitigate mental distress. Public health programmes should pair reproductive rights advocacy with community-based mental health outreach, prioritising settings where legal or financial barriers are steepest.

The wide range of depressive symptom prevalence we observed (5–25%) is mirrored in recent U.S. population-based work showing that mental distress risk rises only where abortion access is actively curtailed. In a 2024 multistate cohort, exposure to highly restrictive legislation predicted a 35% relative increase in antenatal or postpartum depression independent of income, parity, or trauma history (McKetta et al., 2024). These data reinforce our interpretation that the macro-policy environment, rather than procedure frequency per se, calibrates psychological outcomes; jurisdictions with liberal laws report background-level distress even among women with three or more abortions.

Stigma remains an under-recognised pathway. Qualitative interviews from the United Kingdom illustrate how the very label “repeat abortion” can become a “moral scarlet letter,” prompting secrecy, social withdrawal, and internalised shame (Hoggart et al., 2017). Our review’s finding that unwanted disclosure predicted higher DASS-21 scores dovetails with those narratives: women who must justify multiple procedures to sceptical partners or clinicians shoulder a double burden of logistical stress and identity threat. Destigmatising language and trauma-informed counselling, therefore, are not ancillary niceties but core components of post-abortion mental health care.

Interpersonal power dynamics also shape risk trajectories. A 2025 CDC analysis of 29,000 postpartum respondents found that 5% conceived after a partner blocked contraception use; this subgroup accessed fewer prenatal visits and reported poorer mental health scores at six months (D’Angelo et al., 2025). Such findings align with our meta-narrative synthesis where low partner support and economic coercion independently amplified depressive odds. Integrating routine intimate partner violence screening and offering discreet LARC options at the point of care could mitigate both repeat pregnancy and psychological sequelae.

Adolescents with pre-existing psychiatric morbidity constitute another vulnerable stratum. In a 2024–2025 prospective U.S. hospital series, 78% of psychiatric inpatient girls expressed interest in contraception yet only 18% initiated a method before discharge; linkage to dedicated counselling halved six-month repeat-pregnancy risk and improved PHQ-9 trajectories (Underwood et al., 2024). Our review contained no adolescent-specific cohorts, underscoring a research gap. Embedding contraceptive services within behavioural health settings—and vice versa—may be pivotal for breaking the cycle of untreated mental illness, contraceptive inequity, and repeat abortion among youth.

Finally, cross-regional evidence underlines the universality of structural barriers. Among Chinese teenagers presenting for post-abortion care, worries about contraceptive side effects and provider judgement curtailed LARC uptake to 32% despite national guidelines (Jin et al., 2024). In Cape Town, adolescent girls cited clinic wait-times, stock-outs, and confidentiality concerns as drivers of discontinuation and repeat pregnancy (Tolla et al., 2024). These patterns echo our synthesis: when health systems prioritise privacy, method mix, and youth-friendly hours, both contraceptive continuity and psychological well-being improve. Global programmes should therefore pair rights-based legal reforms with context-tailored service innovations to achieve sustained mental health gains after abortion.

QoL outcomes were infrequently reported and measured with heterogeneous tools, limiting inference. Where assessed, denied or heavily delayed abortions corresponded to modest decrements on satisfaction-with-life and functioning scales. Because QoL integrates health, social role participation, and financial security, it may be more sensitive to structural constraints (travel, cost, legal risk) than to procedure count. Future evaluations should pre-specify one generic instrument (e.g., EQ-5D-5L with utility weights) alongside mental health scales to enable cost-utility analyses and service delivery comparisons.

Stronger evidence (moderate certainty) demonstrated (a) increased distress following abrupt policy restrictions (e.g., SB8), supported by quasi-experimental difference-in-differences and multi-level analyses; and (b) associations between stigma-related experiences (unwanted disclosure, low partner support) and higher depressive symptom scores in clinic cohorts using validated instruments. More tentative evidence (low to very low certainty) demonstrated (a) a modest association between ≥3 abortions and later-life depressive symptoms in a specific Chinese cohort, potentially modified by socioeconomic indications; and (b) generic QoL decrements after denial or delay of abortion, reported in few studies with heterogeneous instruments.

Future work should (i) use standardised mental health and QoL instruments across settings (e.g., PHQ-9/GAD-7/PSS plus EQ-5D-5L or PROMIS Global-10) with harmonised cut-points; (ii) adopt prospective longitudinal designs with ≥3 assessment waves to capture trajectories; (iii) apply causal-inference strategies (e.g., difference-in-differences around policy shocks; inverse-probability weighting for confounding by adversity; pre-specified DAGs); (iv) oversample adolescents, LGBTQIA+ populations, and those experiencing partner coercion; and (v) to advance comparability, future studies should adopt a core outcome set for psychological well-being after abortion—PHQ-9, GAD-7, PSS-10, and one generic QoL instrument (EQ-5D-5L or WHOQOL-BREF)—assessed at standardised intervals (baseline/entry, ~6–12 weeks, and 6–12 months). Reporting should include minimally important differences, ceiling/floor checks, and measurement invariance across language and policy contexts.

### 4.2. Limitations

Our synthesis is constrained by moderate heterogeneity and reliance on self-reported abortion histories, potentially introducing recall bias. QoL instruments were seldom used, limiting the evaluation of broader well-being. Only English-language and selected non-English studies were included, possibly excluding valuable regional data. Population coverage was incomplete: no included study provided subgroup analyses for adolescents or LGBTQIA+ individuals experiencing repeat abortion. Given distinct stigma pathways and contraceptive needs in these groups, dedicated sampling frames and inclusive measures (e.g., gender identity items, partner coercion metrics) are warranted. Finally, meta-analysis was precluded by outcome variability; the results should thus be interpreted narratively.

## 5. Conclusions

Repeat induced abortion alone is not a reliable predictor of poor mental health. Instead, structural and interpersonal barriers to modern contraception—and the stigma they engender—drive much of the observed psychological burden. Policies expanding reproductive autonomy, reducing disclosure pressure, and enhancing social support are pivotal for safeguarding women’s mental health and quality of life.

## Figures and Tables

**Table 1:** Characteristics of included studies.

Author (Year)	Country	Design	Sample (n)	Population/Repeat Abortion Definition	Mental Health Instrument	Contraception/Access Variables
(Li et al., 2024)	China	Obs. cohort	9991	Post-menopausal women; ≥3 abortions vs. 0	PHQ-9, GAD-7	Socioeconomic vs. medical indication
(Rajkumar, 2022)	Global (197 countries)	Ecological	NR (nation-level)	Child-bearing-age women; policy shift	GBD prevalence modelling	Legal access index (2009, 2017)
(Jamali et al., 2025)	Iran	Cross-sectional	250	First-trimester seekers; prior abortions recorded	PHQ-4, PSS	Social support, cost, previous abortion
(Biggs et al., 2023)	USA (4 clinics)	Cross-sectional	746	Current abortion seekers; repeat history 27%	DASS-21	Unwanted disclosure, travel, cost
(Biggs et al., 2017)	USA (Turnaway)	Longitudinal cohort	956	Near-limit vs. denied abortion; repeat subgroup	BSI, Rosenberg SE, SWLS	Gestational limit, distance
(Liu et al., 2023)	USA (BRFSS)	Multi-level	159,353 (weighted)	Women 18–49; state-level hostility score	Frequent mental distress (BRFSS)	Composite reproductive rights score
(Lee et al., 2025)	Texas, USA	Quasi-exp. DID	79,609	Females 18–44; SB8 exposure	Frequent mental distress	SB8 legal ban
(Kotta et al., 2018)	India	Cross-sectional	230	Hospital presenters; gestational age > 12 weeks	GHQ-12, IES-R	Socio-demographics, cost

Abbreviations: Obs. cohort—observational cohort; Ecological—country-level ecological design; NR—not reported; PHQ-9—Patient Health Questionnaire-9; GAD-7—Generalised Anxiety Disorder-7; PSS—Perceived Stress Scale; DASS-21—Depression, Anxiety and Stress Scale-21; BSI—Brief Symptom Inventory; Rosenberg SE—Rosenberg Self-Esteem Scale; SWLS—Satisfaction With Life Scale; GHQ-12—General Health Questionnaire-12; IES-R—Impact of Event Scale-Revised; BRFSS—Behavioral Risk Factor Surveillance System; SB8—Texas Senate Bill 8; DID—difference-in-differences analysis.

**Table 2:** Prevalence of psychological outcomes.

Author (Year)	Depression (%)	Anxiety (%)	Stress/Distress (%)	QoL Score	Time-Point
(Li et al., 2024)	5.54	8.27	NR	NR	Mean age 60 years
(Rajkumar, 2022)	NR	NR	NR	NR	Cross-sectional 2010, 2019
(Jamali et al., 2025)	15.6	NR	18.8	NR	Entry to service
(Biggs et al., 2023)	NR (β 0.62↑)	NR (β 1.79↑)	NR (β 1.80↑)	NR	Within 7 days
(Biggs et al., 2017)	NR	Near-limit baseline 0.57 vs. denied 2.29	NR	Life satisfaction −0.41	1 week and 5 years
(Liu et al., 2023)	NR	NR	FMD 12.3	NR	BRFSS 2018
(Lee et al., 2025)	NR	NR	FMD 21.9 (post-SB8)	NR	2022
(Kotta et al., 2018)	24.8	31.2	27	NR	1-month median

Abbreviations: NR—not reported; Depression/Anxiety/Stress (%)—percentage of participants meeting instrument-specific cut-offs; FMD—frequent mental distress (BRFSS case definition of ≥14 mentally unhealthy days in the past month); QoL—generic quality of life score; β—unstandardised regression coefficient; time-point denotes assessment moment specified in each study (e.g., baseline, follow-up).

**Table 3:** Factors associated with poorer mental health/QoL outcomes.

Author (Year)	Barrier/Factor	Effect Measure	95% CI	*p*
(Li et al., 2024)	≥3 abortions	OR 1.37	1.13–1.67	<0.01
(Li et al., 2024)	Abortion for socioeconomic reasons	OR 1.34	1.08–1.66	0.02
(Biggs et al., 2023)	Unwanted disclosure	β 0.62 (Depression)	0.28–0.95	<0.001
(Biggs et al., 2023)	Low support from partner	β 0.67 (Depression)	0.16–1.18	0.01
(Jamali et al., 2025)	Low social support	OR 2.05	NR	0.034
(Jamali et al., 2025)	Previous abortion	OR 2.50	NR	0.001
(Liu et al., 2023)	1 SD ↑ reproductive rights score	OR 0.95 (Distress)	0.91–0.99	0.04
(Lee et al., 2025)	SB8 implementation	ΔFMD + 6.8 pp	3.0–10.6	<0.001

Abbreviations: OR—odds ratio; β—unstandardised regression coefficient; ΔFMD—absolute change in frequent mental distress prevalence; SD—standard deviation; pp—percentage points; CI—confidence interval; *p*—two-sided probability value; SB8—Texas Senate Bill 8; reproductive rights score refers to standardised state-level composite used by Liu et al.; QoL—quality of life.

**Table 4:** Study-level risk-of-bias summary.

Study (Year)	Design	Tool	Selection/Sampling	Exposure/Outcome Measurement	Confounding Control	Reporting Bias	Overall
(Biggs et al., 2017)	Prospective cohort	NOS	Low	Low (validated scales)	Low (pre-specified covariates)	Low	Low
(Lee et al., 2025)	Quasi-experimental (DiD)	ROBINS-I	Low	Low	Moderate (parallel trends assumption)	Low	Moderate
(Liu et al., 2023)	Multi-level cross-sectional	NOS (adapted)	Low	Moderate (BRFSS FMD proxy)	Moderate (state and individual adjusters)	Low	Moderate
(Li et al., 2024)	Observational cohort	NOS	Moderate (age-restricted sample)	Moderate (self-reported lifetime abortions)	Moderate	Low	Moderate
(Biggs et al., 2023)	Cross-sectional clinics	NOS	Low	Low (validated DASS-21)	Moderate (residual stigma confounding)	Low	Moderate
(Jamali et al., 2025)	Cross-sectional	NOS	Moderate (single-site)	Moderate (self-report)	Serious (limited adjustment)	Moderate	Serious
(Kotta et al., 2018)	Cross-sectional	NOS	Moderate	Moderate (GHQ-12/IES-R valid; abortion history self-report)	Serious	Moderate	Serious
(Rajkumar, 2022)	Ecological	ROBINS-I (adapted)	Low	Moderate (GBD modelled outcomes)	Serious (ecological confounding)	Moderate	Serious

Exposure = repeat induced abortion; Outcome = depression/anxiety/stress/QoL; FMD = frequent mental distress.

## Data Availability

No new data were created or analysed in this study.
